# Efficacy and safety of Qishen Yiqi dripping pills as a complementary treatment for Heart Failure: A protocol of updated systematic review and meta-analysis

**DOI:** 10.1097/MD.0000000000024285

**Published:** 2021-01-15

**Authors:** Hui Guan, Guohua Dai, Lili Ren, Wulin Gao, Haoran Fu, Zepeng Zhao, Xin Liu, Jue Li

**Affiliations:** a First College of Clinical Medicine, Shandong University of Traditional Chinese Medicine; b Department of Cardiology, Affiliated Hospital of Shandong University of Traditional Chinese Medicine, Jinan, Shandong Province.

**Keywords:** complementary treatment, heart failure, meta-analysis, qishen yiqi dripping pills

## Abstract

**Background:**

Heart failure (HF) has become a serious global public health issue due to its high incidence, high mortality and extremely low quality of life. According to several clinical trials, Qishen Yiqi Dripping pills (QSYQ) combined with routine western medicine treatment can further enhance the curative effect of HF patients. However, most of the trials are small in sample size and poor in quality, which can only provide limited evidence-based medicine. The existing systematic reviews of efficacy and safety has provided evidence for the clinical application of QSYQ to a certain extent, but there are still 3 major defects. Here, we will perform a systematic review and meta-analysis that include the randomized clinical trial (RCT) of CACT-IHF, apply meta-regression and subgroup analysis to cope with multiple confounding factors, and add the clinical efficacy standards of TCM, all-cause death and readmission rates as reliable efficacy evaluation indicators. The purpose of this study was to rigorously evaluate the clinical efficacy and safety of QSYQ in the complementary treatment of HF with a well-designed systematic review and meta-analysis.

**Methods:**

Following the strict search strategy, 9 databases will be searched to ensure a comprehensive search. We search the database from the establishment until November 30, 2020. This study will include RCTs of QSYQ in HF patients’ complementary treatment. Two searchers will independently draft and carry out the search strategy, and the third member will further complete it. Two members independently screen literature, extract data and cross-check, and solve different opinions through discussion or negotiation with the third member. The risk bias will be evaluated based on Cochrane tool of risk of bias. Meta-regression and subgroup analysis are used to check and deal with the heterogeneity. The data analysis will be conducted by the statistical software Stata 16.0.

**Results:**

The results of this research will be delivered in a peer-reviewed journal.

**Conclusion:**

This study expects to provide credible and scientific evidence for the efficacy and safety of QSYQ in HF's complementary treatment, and at the same time provide a convenient and effective choice for decision-makers and patients.

**Protocol registration number:**

INPLASY 2020120106.

**Ethical approval:**

Since this study is on the basis of published or registered RCTs, ethical approval and informed consent of patients are not required.

## 1 Introduction

### 1.1 Description of the condition

Heart failure (HF) is the common end-stage of various cardiovascular diseases, which brings heavy economic burdens to families and society. The progress of modern medical conditions improves the survival rate of patients with cardiovascular diseases, which will make the prevalence rate of HF continue to rise in the future. At the same time, the mortality rate of patients with severe HF reached more than 70% within 5 years.^[1,2]^ In addition, patients with HF have the risk of aggravating symptoms, such as dyspnea, fatigue, oedema and decreased exercise ability, which makes patients very painful.^[3,4]^ HF have become a serious public health problem worldwide owing to its high incidence, high mortality, and extremely low quality of life.^[5]^ It is urgent to find a complementary therapy that can enhance the quality of life and further effectively reduce the rates of readmission and mortality.

### 1.2 The complementary intervention of Qishen Yiqi dripping pills (QSYQ)

QSYQ is a small dripping pill made from 4 kinds of traditional Chinese medicine (TCM): Astragalus, Salviae, Panax notoginseng, and Dalbergia wood oil.^[6]^ This Oral Chinese patent medicine is a patented drug (drug approval number: Z20030139), which is produced by the Tasly Pharm (Tianjin, China). It has been widely used in the treatment of coronary heart disease and angina pectoris since its inception in 2003. The main indications include chest distress, chest pain, shortness of breath, fatigue, palpitation, spontaneous sweating and poor complexion. Clinical exploratory and pharmacological researches have confirmed that the drug can improve myocardial energy metabolism, enhance left ventricular ejection fraction, increase exercise volume and reduce readmission rate. Because of its advantages in the treatment of HF patients in the early and mid-stage clinical, it was officially approved for clinical trials that treating HF by the China Medical Products Administration Agency in January 2020.^[7]^ The existing systematic review (SRs) provide evidence for clinical application of QSYQ to a certain extent, but there are still several shortcomings. For instance, it did not deal with clinical multiple confounding factors; All-cause death and readmission rates, which reflect long-term prognosis, are not included in efficacy evaluation; The latest high-quality clinical study was not included.^[8,9]^ Recently, the randomized controlled trial (RCT) of QSYQ treating HF involving the largest sample of 640 patients in 24 centers around the world was published. The study adds composite clinical outcomes (including all-cause mortality within 12 months, emergency treatment/ readmission owing to heart failure, cardiogenic shock, etc) as evaluation indicators for the first time.^[10]^ There is no doubt that this trial will have an unprecedented impact on the clinical evidence-based evaluation of QSYQ. However, none of a SR includes this trial in statistical analysis, not even the latest meta-analysis (MA) that includes 85 RCTs.^[11]^

### 1.3 Objectives

This study will include the latest RCT research, deal with multiple confounding factors by meta-regression and subgroup analysis, add all-cause mortality, readmission rate, and the clinical efficacy standards of TCM as evaluation indicators. The purpose of this study is to assess the clinical efficacy and safety of QSYQ in the complementary treatment of HF. This study aims to comprehensively evaluate the clinical efficacy and safety of QSYQ in the complementary treatment of HF with a well-designed SR and MA.

## 2 Methods

### 2.1 Study registration

This MA will be guided by the preferred reporting items for systematic review and meta-analysis protocols (PRISMA-P) 2015 and Cochrane Handbook for Systematic Reviews of Interventions.^[12,13]^ The protocol has been registered on the INPLASY website, and the registration number is INPLASY 2020120106 (URL= https://inplasy.com/inplasy-2020-12-0106/).

### 2.2 Eligibility criteria

Eligibility criteria will follow the principles of the PICOS, including the following:^[14]^

#### 2.2.1 Study of type

This study only includes the published or ongoing RCTs of QSYQ for HF. These studies will be excluded:

(1) those that cannot reflect the clinical efficacy of QSYQ;
(2) The sample size of the QSYQ group/control group is less than 40 cases. If the studies are similar, only the studies with the highest quality or the largest sample size will be retained.

#### 2.2.2 Participants

Patients diagnosed with HF and NYHA classification II-IV will be included in the study according to any diagnostic criteria. Patients undergoing cardiac resynchronization therapy, coronary artery bypass surgery, or non-cardiovascular events (such as malignant tumors, mental illness, or severe liver and kidney insufficiency) will be excluded.

#### 2.2.3 Interventions and comparators

Both the QSYQ and control groups receive routine western medicine treatment recommended by the guidelines, including diuretics, ACEI/ARB, digitalis drugs, beta-blockers, aldosterone receptor antagonists, nitrate drugs, etc. The QSYQ group is treated with QSYQ based on the control group. The routine treatment in each RCT does not need to be consistent, but the only difference between the QSYQ and control groups should be whether to apply QSYQ. Besides, neither group takes any drugs that may interfere with the evaluation indicators.

#### 2.2.4 Outcomes

Primary efficacy evaluation indicators contain effective rate, all-cause mortality, emergency treatment/readmission due to HF, and other cardiovascular outcomes.

Secondary efficacy evaluation indicators contain the clinical efficacy standards of TCM, 6-minute walk test, New York Heart Association classification, left ventricular ejection fraction, brain natriuretic peptide/N-terminal pro-brain natriuretic peptide, and other alternative indicators.

Safety indicators include skin itching or rash, nausea, vomiting, dizziness and other adverse events.

According to China's Guiding Principles for Clinical Research on New Drugs of TCM, the clinical efficacy standards are as follows.

(1) Significantly effective: after treatment, the primary and secondary symptoms basically or completely disappeared, and the quantitative score of syndromes was 0 or decreased by 70%.
(2) Effective: after treatment, the quantitative score of syndromes decreased by 30%.
(3) Invalid: after treatment, the quantitative score of syndromes decreased by less than 30%.
(4) Aggravation: the quantitative score exceeds the pre-treatment score.

### 2.3 Data sources

#### 2.3.1 Electronic search

We will search PubMed/ MEDLINE, Web of Science, EMBASE, Cochrane Library, CNKI, Wanfang Database, VIP Database, Chinese Scientific Journal Database, Chinese Biomedical Literature Database.^[15]^ The search time is from the database establishment until November 30, 2020. We will also search for ongoing RCTs, such as trials registered and conducted on the WHO International Clinical Trial Registration Platform or Chinese Clinical Trial Registry.

#### 2.3.2 Other sources of data

Besides searching the electronic database, we also perform the manual search, reference tracking and retrieval by the search engine.

#### 2.3.3 Search strategy

Heart failure, Qishen Yiqi Dripping Pills, randomized controlled trials and their synonyms are used as search terms. The search strategy is a combination of free text words and Medical Subject Headings, and we will adjust the search strategy according to different search strategies. Two searchers will independently draft and carry out the search strategy, and the third member will further complete it. Table 1 shows PubMed's detailed search strategy.

**Table 1:** Search strategy for PubMed.

No.	Search item
#1	Heart Failure[Mesh]
#2	Heart Failure[Title/Abstract] OR Cardiac Failure[Title/Abstract] OR Heart Decompensation[Title/Abstract] OR Decompensation, Heart[Title/Abstract] OR Heart Failure, Right-Sided[Title/Abstract] OR Heart Failure, Right Sided[Title/Abstract] OR Right-Sided Heart Failure[Title/Abstract] OR Right Sided Heart Failure[Title/Abstract] OR Myocardial Failure[Title/Abstract] OR Congestive Heart Failure[Title/Abstract] OR Heart Failure, Congestive[Title/Abstract] OR Heart Failure, Left-Sided[Title/Abstract] OR Heart Failure, Left Sided[Title/Abstract] OR Left-Sided Heart Failure[Title/Abstract] OR Left Sided Heart Failure[Title/Abstract]
#3	#1 OR #2
#4	Qishen Yiqi Dripping Pills[Mesh]
#5	Qishen Yiqi Dripping Pills[Title/Abstract] OR Qishen Yiqi Dripping Pill [Title/Abstract] OR Qishen Yiqi DropPill[Title/Abstract] OR Qishen Yiqi [Title/Abstract] OR Qishen Yiqi droplet[Title/Abstract] OR Qishen Yiqi Pills[Title/Abstract]
#6	#4 OR #5
#7	randomized controlled trial [Publication Type]
#8	controlled clinical trial [Publication Type]
#9	randomized [Title/Abstract]
#10	randomly [Title/Abstract]
#11	#7 OR #8 OR #9 OR #10
#12	#3 AND #6 AND #11

### 2.4 Literature screening and data extraction

Two members will use the Endnote X9 to screen literature, extract data and cross-check independently. Resolving inconsistent opinions through discussion or negotiation with the third member. For screening Literature, first read the title, exclude irrelevant literature, then read the abstract and full text, and decide whether to include. If necessary, we will contact the author of the original study by e-mail or telephone to obtain missing but essential information for this study.^[16]^ The data is extracted as follows:

Basic features: title, first author, publication year, country, journal, and so on.
Participant characteristics: age, sex, eligibility criteria, sample size, and so on.
Interventions: dosage, frequency, intervention time, and so on.
Evaluation Indicators: primary and secondary efficacy evaluation indicators, safety indicators.

### 2.5 Statistical analysis

#### 2.5.1 Date analysis

Different effect indicators are chosen according to the data types of evaluation indicators. For binary variables, we will calculate the odds ratio or relative risk and its 95% confidence interval (CI). For continuous variables, we will calculate the mean difference or standardized mean difference and its CI. The statistical analysis is performed with the help of stata16.0 software.

#### 2.5.2 Assessment of risk of bias

Seven items are evaluated by the Cochrane Collaboration's bias risk evaluation tool.^[17]^ The items are divided into 3 evaluation grades: low risk of bias (Grade-A), high risk of bias (Grade-C) and uncertain whether there is bias (Grade-B). All 7 items are Grade-A means the risk of bias is very low. If there are Grade-B items and no Grade-C item, it demonstrates that the study has a moderate risk of bias. The existence of Grade-C item indicates that the study has a high risk of bias.

#### 2.5.3 Heterogeneity analysis

The possible differences in age, race, clinical research quality, drug dosage and other clinical factors may lead to heterogeneity. Therefore, we should analyze the sources of heterogeneity and explore the influence of these factors on the total effect and its degree. The heterogeneity test adopts the *I*^2^ test and quantitative analysis. When *I*^2^ < 50%, the heterogeneity is not obvious, we will use the fixed effects model; when *I*^2^≥50%, the heterogeneity is significant, and the random effects model is used.^[18]^ Meta-regression and subgroup analysis are applied to discover the source of heterogeneity and deal with it. First of all, a meta-regression model is established to screen the influencing factors of heterogeneity. Then a subgroup analysis considering this influencing factor is carried out to compare the changes of heterogeneity before and after.^[19]^

#### 2.5.4 Assessment of Publication bias

If the evaluation indictor is a binary variable, the harbor test can be used to explore publication bias. Correspondingly, if the evaluation indictor is a continuous variable, the egger test is performed. The results are expressed by effect size and 95% CI.

#### 2.5.5 Evaluation of evidence quality

The GRADE method is adopted to assess the quality of evidence and the strength of recommendations. The quality of evidence can be classified into 4 levels: high, medium, low and extremely low.^[20]^

## 3 Discussion

Considering the high incidence, high mortality, high cost and low quality of life of HF, it is urgent to find a complementary therapy that can improve the quality of life and further effectively decrease the rates of readmission and mortality. Clinical studies show that oral Chinese patent medicine combined with routine treatment can improve the clinical symptoms, exercise endurance, quality of life, laboratory examination, physical examination of HF patients.^[21]^ In addition, QSYQ can lower the readmission rate of HF patients.^[22]^ Experimental studies show that in the aspect of inflammation, QSYQ can relieve ventricular remodeling by inhibiting the inflammatory factor IL-6 TNF-α and related signal transduction pathway proteins. In the aspect of activation of the RAAS system, QSYQ can inhibit the expression level of AngII in myocardial tissue, down-regulate the AT1 receptor, increase the content of the protective receptor AT2, and up-regulate the expression level of PGC1-α to alleviate ventricular remodeling. In general, QSYQ can act on multiple links and multiple targets, to prevent and delay ventricular remodeling, achieve the effect of reducing the recurrence and slow the course of HF.^[23–25]^

In recent years, the RCTs of QSYQ in the complementary treatment of HF are on the rise, but the evidence level of MAs is low owing to the small sample sizes and low quality of most studies. The latest updated MA includes 85 studies, with an average sample size of 85 cases. The sample sizes of 50.5% studies are less than 100 cases, with a minimum sample size of 30 cases and a maximum sample size of 212 cases.^[26]^ The QSYQ treatment course is divided into 1 to 4 weeks, 5 to 8 weeks, ≥9 weeks, and the number of corresponding studies is 29, 24, and 32 respectively.^[11]^ For HF patients, it takes a certain period to receive TCM treatment before it can be effective. Moreover, TCM has advantages in the long-term prognosis of patients through overall adjustment. However, current studies have too short follow-up time, and ignore efficacy evaluation indicators such as readmission rate and all-cause death, which makes the advantages of TCM unable to be highlighted.

At present, clinicians haven’t reached a consensus on the clinical application of QSYQ, and there lacks in uniform and standards. Recently, Mao Jingyuan team from China published an article on the RCT of QSYQ complementary treatment for HF. The study includes 640 patients in 24 centers around the world and becomes the most influential RCT with the highest sample content for QSYQ.^[10]^ Up to now, there has not been a SR and MA to include this study. Therefore, it is essential to include this study, at the same time, restricting low-level studies, and conducting an updated, high-quality SR and MA to provide evidence-based medical evidence for QSYQ in the treatment of HF.

If studies with small sample sizes are artificially excluded, there may be a selection bias, which is the limitation of the research. But in comparison, the damage caused by low-quality studies may be greater, and we will also take measures to reduce the impact of selection bias. Anyway, we expect this study will provide strong evidence of QSYQ in the complementary treatment of HF than previous MAs.

## Author contributions

**Conceptualization:** Hui Guan, Lili Ren, Guohua Dai

**Data curation:** Hui Guan, Lili Ren, Haoran Fu

**Formal analysis:** Hui Guan, Zepeng Zhao

**Funding acquisition**: Guohua Dai

**Methodology:** Hui Guan, Wulin Gao, Xin Liu

**Project administration**: Guohua Dai

**Writing – original draft**: Hui Guan, Lili Ren

**Writing – review & editing**: Hui Guan, Jue Li

## Abbreviations

Abbreviations: HF = heart failure, MA = meta-analysis, QSYQ = Qishen Yiqi Dripping pills, RCT = randomized controlled trial, SR = systematic review.
